# Exposure to persons with symptoms of respiratory or gastrointestinal infection and relative risk of disease: self-reported observations by controls in a randomized intervention trial

**DOI:** 10.1186/s13063-015-0691-4

**Published:** 2015-04-17

**Authors:** Tapani Hovi, Jukka Ollgren, Jaason Haapakoski, Ali Amiryousefi, Carita Savolainen-Kopra

**Affiliations:** National Institute for Health and Welfare (THL), Department of Infectious Diseases, Viral Infections Unit, PO Box 30, FIN-00271 Helsinki, Finland; Department of Mathematics and Statistics, University of Helsinki, Helsinki, Finland

**Keywords:** Exposure, Respiratory infection, Gastrointestinal infection, Occupational health

## Abstract

**Background:**

Little is known about the quantitative relationships between a self-recognized exposure to people with symptoms of respiratory (RTI) or gastrointestinal tract infection (GTI) and subsequent occurrence of homologous symptoms in the exposed person.

**Methods:**

Adult office employees, controls in an intervention trial, reported weekly own symptoms of RTI or GTI and exposures to other persons with similar symptoms. To ascertain the reliability of the self-reported data, the participants received both in-advance training and repeated instructions in the weekly Email requests for reports. The relationship of self-reported exposures to self-reported homologous symptoms during the same or the following week was analyzed including, in the statistical models, cluster effects and longitudinal aspects in the data, seasonality, and cluster-specific baseline values.

**Results:**

Altogether 11,644 weekly reports were received from 230 participants during the 16-month duration of the study. The mean age of the reporters was 42.9 years (standard deviation 11.1 years), and the female/male ratio 157/68 (for 5 participants this information was not available). A reported exposure to RTI was associated with an almost 5-fold higher relative risk for a reported homologous infection during the same week (4.9; 95% confidence interval (CI) 4.0 to 5.9), and with a 3-fold risk during the following week (3.3; CI 2.8 to 3.8). For GTI the corresponding figures were 15.1 (CI 10.4 to 21.8) and 4.3 (CI 3.1 to 5.8), respectively. On the other hand, for 24% of the designated RTI episodes, a homologous exposure had been reported during neither the same nor the preceding week. For GTI this figure was even greater (40%). For both RTI and GTI, weeks with a reported exposure were more frequent outside the workplace than only at the workplace (434 versus 262, and 109 versus 41, respectively).

**Conclusion:**

A reported exposure to persons with obvious symptoms of RTI or GTI significantly increased the relative risk of reported homologous infection in the exposed adult persons. Yet, a substantial part of reported designated RTI and, especially, GTI episodes occurred without a reported exposure during the same or the previous week.

**Trial registration:**

ClinicalTrials.gov with an identifier of NCT00821509 (12 March 2009).

## Background

The most common infectious diseases are caused by viral agents and occur in the respiratory [1] and gastrointestinal tract [2]. In addition to enhanced hand hygiene [3-7], social distancing; that is avoidance of public gatherings, crowded public transport vehicles and shopping centers, close contacts with symptomatic persons, and so on, has been suggested as a means of limiting virus transmission during epidemics, including influenza pandemics [3]. However, it is not clear how feasible social distancing could be in everyday life. One can decide about staying away from public gatherings during free time, whereas using public transport to and from the workplace, and attending scheduled meetings at work may be unavoidable. Likewise, while it might be possible at work to stop shaking hands and to avoid other close contacts with colleagues during epidemics, parents cannot avoid close contact with their sick children. The potential effectiveness of social distancing, and especially avoidance of other persons with symptoms of respiratory tract infection (RTI) or gastrointestinal tract infection (GTI), is partly jeopardized by the fact that virus shedding may already have started before onset of the symptoms [8] and, on the other hand, continue after cessation of the symptoms. For instance, contrary to previous beliefs, norovirus-infected individuals frequently shed the virus for several days after the gastroenteritis symptoms have ended [9,10]. Furthermore, virus-infected persons may remain completely symptomless and still be able to shed the virus.

Very little seems to be known in the literature about self-experienced exposure to other people with RTI or GTI symptoms, and consequent emergence of homologous symptoms in the observer. While this association is expected, a clear documentation of its existence might help to design hand hygiene and behavioral instructions for people in order to limit virus transmission during epidemics.

We have conducted a 16-month cluster-randomized 3-arm occupational health intervention trial, the STOPFLU Study [11], and reported earlier that in the intervention arm executing enhanced hand hygiene with water and soap, the occurrence of self-reported infection episodes decreased by 16.7%, approximately equating to 1 infection episode per person year [12]. The data collection took place through a standardized electronic questionnaire sent weekly via personal Emails. In the STOPFLU Study we also collected, on a weekly basis, data on self-recognized exposures to other persons with respiratory or gastrointestinal disease symptoms. Here, we report the association of the self-reported exposures at work or outside the work with self-reported homologous RTI or GTI symptoms occurring in the reporter during the same week or the following week. This analysis is limited to the participants in the control arm only.

## Materials and methods

### General study design

We studied the efficacy of enhanced hand hygiene on infection episodes and absences from work in office environments in an open, cluster-randomized intervention trial. The study design has been reported in detail previously [11]. The protocol was accepted by the Institutional Review Board of the National Public Health Institute (KTL) (9/2008) and registered at ClinicalTrials.gov (http://clinicaltrials.gov/) with an identifier of NCT00821509 (12 March 2009). In short, a total of twenty-one distinct office work units, later referred to as clusters, were identified in six corporations in the Helsinki Region.Chief physicians of the occupational health clinics serving the staff in each of these corporations evaluated and approved the protocol, after consulting the Health and Safety Committees of the following corporations: Kesko Oyj, Outokumpu Oyj, Outotec Oyj, Nordea Bank Finland Plc, SOK group, and S-Bank**.** In collaboration with the occupational health clinics, volunteers were recruited among the 1,270 employees working in these units, after excluding persons with open wounds or chronic eczema of the hands. This group received a personal Email from the researchers, including an electronic contagion risk survey enquiring about, for example, type of children’s day-care, potential smoking, frequency of work trips, physician-diagnosed chronic diseases, and so on. The reply Emails included a statement of voluntary participation in the study. Only employees who gave this informed consent were enrolled into the study. An arbitrary virus transmission risk score was calculated for each cluster based on the results of the contagion risk survey. The clusters were then ranked according to the score and divided in seven triplets on the basis of the rank. One member of each triplet was finally randomized into each of the three trial arms. According to the protocol, all new employees hired into these work units during the trial, had to be offered the possibility of participating in the study [11].

### Collection of data for self-reported exposures and for infections

All the data collected was based on self-reporting. The participants recorded their infection symptoms and exposures to sick persons on an Internet-based questionnaire, a link to which was sent via Email on Monday mornings [11]. The data collection software was acquired from Digium Enterprises, Espoo, Finland. When replying to the weekly questionnaires the participants reported day by day possible suffering from RTI or GTI symptoms during the previous 7 days, as well as, on a weekly basis, whether they had recognized exposure to other persons with RTI, GTI or both during the week. During the in-advance training as well as in each weekly Email, symptoms typical of acute RTI or GTI were described in detail. An exposure to RTI or GTI was defined as ‘having met people with obvious symptoms of either respiratory or gastrointestinal tract infection', respectively, without stating in writing relevant physical distance or minimal time of contact. Common sense factors influencing transmissibility of infections when meeting somebody with apparent infectious disease were, however, thoroughly discussed in training meetings of the volunteers before starting the interventions as well as during the research nurse’s site visits [11] in order to exclude irrelevant reports. In addition, the participants reported the site of exposure, whether at work, during travel to or from the workplace, at home, or elsewhere during free time, as described earlier in detail [11]. For purposes of the current analysis the reported exposure site data was divided into ‘only at work', ‘only elsewhere', that is not at work, and ‘both at work and elsewhere’ categories. A definition of a week with RTI or GTI infection was based on self-reported symptoms of RTI or GTI, respectively, at least on one day during the week. A week with both reported RTI and reported GTI symptoms was listed in both categories. As reported before [12], a designated RTI (or GTI) episode was a continuum of successive days with reported RTI (GTI) symptoms, respectively. An episode could continue over one or more weekends.

### Data quality considerations and statistical analysis

#### Missing data

The trial lasted for 16 months but weekly reports were not available from all participants throughout the entire period. In order to be able to use generalized estimating equations (GEE) for marginal models without inverse weighting due to missingness [13], the missing data should be missing completely at random (MCAR), which means in a longitudinal data setting covariate dependent missingness. Most of the missing recordings in this study were ‘according to the protocol’: that is anticipated already at the beginning. In one participating corporation, the final identification of the clusters, and thus the onset of reporting, was delayed due to operational reorganization. This resulted in apparently missing data in the corresponding clusters during a few weeks at the beginning. Even longer and variable-length lack of early recordings was associated with new recruits to the study. The protocol did not require regular reporting during holiday weeks, even though this was possible. This contributed to the observed intermittent data missing. Intermittent data missing as such was sparse, occurring in 87% of the participants 3 times or less frequently. The missing weeks represented only 3% of the maximum potential number of weekly reports, and all missingness not related to the factors described above was rare. In the statistical model, however, the follow-up time of all participants, including those who had been recruited after the onset of the trial, had to be taken as the entire study duration, 16 months. From this point of view, data was available for 75% of the theoretical maximum of follow-up person weeks. The missingness was very similar in all three trial arms. We considered that missing data was not affecting the analysis of the outcome of this study.

#### Dropouts

According to the protocol, the participants were allowed to stop reporting without giving a reason. In order to show that the dropout process was MCAR, logistic regression was used to assess the effect of previous outcomes and covariates to the dropout indicator [13]. The dropout processes associated with RTI and GTI seemed to be MCAR (data not shown).

#### Models for statistical analysis

Multilevel mixed-effects logistic regression model using the Stata program package (Stata version 11.2, StataCorp LP, College Station, TX 77845, USA) was fitted for RTI/GTI outcome with cluster-specific and person-level random effects: seasonality, randomization triplet, and homologous observed exposure (as reported for the same or the preceding week) as covariates. It was noted that the standard deviation (SD) of the cluster-specific random effects was about 3.0e-7 for RTI and about 5.0e-9 for GTI, or lower, somewhat depending on covariates used in the mixed effects logistic regression models. While the above considerations are designed for an analysis using the logit link-function in the model, the design effect is expected to be of the same magnitude in the mixed effect log-link binomial regression, for which a corresponding procedure was not available in the Stata program. However, because we wanted to estimate the relative risks and to take the longitudinal effects into account using robust variance estimators, we finally decided to use marginal GEE log binomial regression models in which clustering due to clusters was ignored as being negligible. Also, the computational burden in calculating the predictive means was then reduced significantly. We fitted the GEE-models with working correlation structures (independent/exchangeable/first order auto-regressive) to assess the impact of reported exposures to the RTI/GTI outcome. To choose the best correlation structure to the longitudinal aspect of the data we used the quasi-likelihood model criterion (QIC) and chose covariates by the corrected quasi-likelihood under independence model criterion (QICC). A difference of at least 4 to 8 in the values of QIC (and QICC) was considered to be significant [14]. Most results seemed to be robust with respect to the chosen correlation structure.

## Results

### General observations

Recruitment to the STOPFLU Study started in January 2009, the first weekly reports received were concerning February 2009, and the trial was stopped in May 2010. Originally, 224 persons who had agreed to participate in the trial, were allocated to the control arm. The total staff number in the designated 7 control clusters was 786, ranging from 50 to 160 (median 100) per cluster. The initial numbers of participants in the clusters ranged from 20 (out of 50) to 36 (median 33) [12], while the enrolled proportions ranged from 22 to 40% (median 28%) of the staff. After the onset of the trial, 16 new persons were recruited into the control clusters [12]. Ten out of the 240 participants did not report a single person week, and thus data from 230 persons was analyzed. Seventy-five of them (32.6%) did not send a report for the last study week, and from the point of view of the intervention trial were considered as dropouts. The most common reported reason for discontinuing reporting was quitting working in the study cluster [12]. Information on age and gender was not available for five reporters. Among the remaining 225 persons, the mean and the median ages were 42.9 years (standard deviation, SD 11.1 years), and 43 years (range 21 to 62), respectively. More than two thirds (157/68) were women.

**Figure 1:**
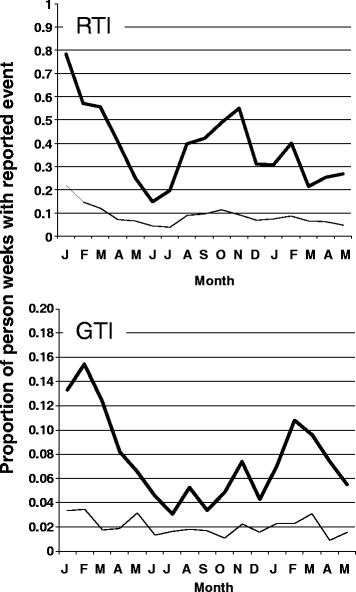
Seasonal variation of reported exposures to (thick line), and own symptoms (thin line) of respiratory infections (RTI), and gastrointestinal tract infections (GTI). Monthly values reflect means of the corresponding weeks. Months are shown by initial letter codes starting from January 2009.

About one third of the weekly reports (3,542 out of 11,644) included a record of a self-recognized exposure to person(s) with infectious disease symptoms, either RTI, GTI, or both. An exposure to RTI was reported in 3,279 person weeks and that to GTI in 767 person weeks, representing proportions of 0.282 and 0.066 of all reported person weeks, respectively. The corresponding predictive margins calculated with the model were 0.282 (confidence interval, CI 0.271 to 0.293), and 0.066 (0.060 to 0.072), respectively. For reference, during the same time 974 reported designated RTI episodes (8.4% of all reported person weeks) and 233 reported designated GTI episodes (2.0%) were evaluable. Seasonal distribution of reported exposures to persons with RTI or GTI symptoms fairly well followed that of the reported RTI and GTI episodes in the responders (Figure 1).

### Association of reported exposures with reported infection symptoms occurring during the same or the following person week

More than a quarter of the reported exposures to RTI (976/3,279) were associated with reported symptomatic homologous disease in the respondent during the same week. The figure was slightly lower during the following week (805/3,279), while almost 40% of the weeks (1,282/3,279) with reported exposure were associated with reported RTI symptoms during either week (Table 1). In contrast, only about one tenth of the weeks without a recognized exposure (904/8,365) were associated with RTI during either the same or the following week. The relative risks for disease in persons reporting an exposure, compared to those without a recognized exposure, was almost 5-fold (risk ratio (RR) 4.9, CI 4.0 to 5.9, *P* < 0.001) during the same week and more than 3-fold during the following week (RR 3.3, CI 2.8 to 3.8, *P* < 0.001).

**Table 1:** **Overall association of reported exposures with reported symptoms of respiratory tract infection (RTI) during the same or the following week**

**Reported exposure to RTI**		**Reported homologous symptoms: Person-week in relation to exposure/Number and proportion of weeks with symptoms of RTI infection/Statistical analysis**					
**Record**	**Number of weeks**	**Same week**		**Following week**		**Either week**	
		**Number**	**Proportion/predictive margin** ^**a**^	**Number**	**Proportion/predictive margin** ^**a**^	**Number**	**Proportion/predictive margin** ^**a**^
Yes	3,279	976	0.298/0.290 (CI^b^ 0.254 to 0.324) RR^c^(exposed/unexposed) = 4.9 (CI 4.0 to 5.9), *P* < 0.001	805	0.246/0.249 (CI 0.215 to 0.282) RR(exposed/unexposed) = 3.3 (CI 2.8 to 3.8), *P* < 0.001	1,282	0.391/0.392 (CI 0.352 to 0.431) RR(exposed/unexposed) = 3.4 (CI 3.0 to 3.9), *P* < 0.001
No	8,365	494	0.059/0.060 (CI 0.050 to 0.069)	594	0.071/0.076 (CI 0.066 to 0.085)	904	0.108/0.115 (0.101 to 0.129)

^a^GEE logistic regression model using Stata was fitted for RTI outcome with seasonality, triplet and homologous reported exposure (same or previous week) as covariates. The working correlation matrix was ‘independent’ and chosen by the quasi-likelihood model criterion (QIC).

^b^95% confidence interval.

^c^RR, risk ratio.

In the next step we reanalyzed the exposure –RTI association after breaking down the exposure data according to the site of exposure: ‘only at work', ‘only elsewhere’, and ‘both at work and elsewhere’ categories. Somewhat more exposures had been recorded outside the work than ‘only at work’ (1,448 versus 1,068). The relative risk of reported RTI symptoms during the same week associated with reported exposure outside the work was significantly higher than that associated with an exposure reported ‘only at work’ (RR 1.23, CI 1.09 to 1.45 (Table 2). During 23.3% of the reported exposure-positive person weeks the exposure had been recognized ‘both at work and elsewhere’. This double-site exposure was associated with an increased rate of reported infections during both the same and the following week (Table 2), with RRs of 1.3 (CI 1.1 to 1.5) and 1.3 (CI 1.1 to 1.6), respectively, between the double-site exposure and the exposure only-not-at-work. For infections reported occurring during either week the observed slightly increased risk was not statistically significant (RR 1.12, CI 0.96 to 1.28).

**Table 2:** **Association of reported homologous exposure with reported respiratory tract symptoms during the same or the following week including comparisons between sites of exposure**

**Reported exposures**		**Reported symptoms: Person-week in relation to exposure/Number and proportion of weeks with symptoms/Statistical analysis**					
**Site**	**Number of weeks**	**Same week**		**Following week**		**Either week**	
		**Number**	**Proportion/predictive margin** ^**a**^	**Number**	**Proportion/predictive margin** ^**a**^	**Number**	**Proportion/predictive margin** ^**a**^
Only at work (W)	1,068	262	0.245/0.236 (CI^b^ 0.198 to 0.274) RR(W/0)^c^ = 4.0 (CI 3.2 to 4.9), *P* < 0.001	221	0.207/0.203 (CI 0.169 to 0.237) RR(W/0) = 2.7 (CI 2.2 to 3.2), *P* < 0.001	359	0.336/0.334 (CI 0.287 to 0.382) RR(W/0) = 2.90 (CI 2.5 to 3.4), *P* < 0.001
Only elsewhere (E)	1,448	434	0.300/0.291 (CI 0.251 to 0.331) RR(E/0) = 4.9 (CI 4.0 to 6.0), *P* < 0.001 RR(E/W) = 1.231 (CI 1.1 to 1.4)	366	0.253/0.221 (CI 0.191 to 0.251) RR(E/0) = 2.9 (CI 2.5 to 3.4), *P* < 0.001 RR(E/W) = 1.1 (CI 0.90 to 1.28)	584	0.403/0.404 (CI 0.358 to 0.449) RR(E/0) = 3.5 (CI 3.2 to 4.1), *P* < 0.001 RR(E/W) = 1.2 (CI 1.0 to 1.4)
Both (WE)	763	280	0.367/0.369 (CI 0.302 to 0.435) RR(WE/0) = 6.2 (CI 4.9 to 7.8), *P* < 0.001	218	0.286/0.296 (CI 0.231 to 0.360) RR(WE/0) = 3.9 (CI 3.2 to 5.0), *P* < 0.001	339	0.444/0.453 (CI 0.384 to 0.521) RR(WE/0) = 3.9 (CI 3.3 to 4.7), *P* < 0.001

^a^GEE logistic regression model using Stata was fitted for RTI outcome with seasonality, triplet and homologous reported exposure (same or previous week) as covariates. The working correlation matrix was ‘independent’ and chosen by the quasi-likelihood model criterion (QIC).

^b^95% confidence interval.

^c^RR, risk ratio, where 0 = No exposure from Table 1.

The number of person weeks with a reported exposure to GTI was only one fourth of that for RTI (767 versus 3,279), and the proportion of weeks with exposure-associated own-reported GTI symptoms was also somewhat lower than the corresponding figure for RTI (0.21 versus 0.30 for the same week). Yet, the relative risk of GTI disease in the exposed persons compared to that in the unexposed persons was much higher, especially during the same week (15.1, CI 10.4 to 21.8), as compared to corresponding risk figures of RTI (4.9, CI 4.0 to 5.9). Reported symptoms of GTI during or immediately after a week without a reported homologous exposure occurred at predictive margins of only 0.014 (CI 0.009 to 0.019) and 0.022 (CI 0.016 to 0.028), respectively (Table 3). The number of reported infections during the same week was more than double compared to that of the week following the reported exposure (159 versus 67).

**Table 3:** **Overall association of reported exposures with symptoms of gastrointestinal infection (GTI) during the same or the following week**

**Reported exposure to GTI**		**Reported homologous symptoms: Person-week in relation to exposure/Number and proportion of weeks with GTI symptoms/Statistical analysis**					
**Record**	**Number of weeks**	**Same week**		**Following week**		**Either week**	
		**Number**	**Proportion/predictive margin** ^**a**^	**Number**	**Proportion/predictive margin** ^**a**^	**Number**	**Proportion/predictive margin** ^**a**^
Yes	767	159	0.207/0.213 (CI^b^ 0.17 to 0.26) RR^c^(exposed/unexposed) = 15.1 (CI 10.4 to 21.8), *P* < 0.001	67	0.087/0.0931 (CI 0.066 to 0.120) RR(exposed/unexposed) = 4.3 (CI 3.1 to 5.8), *P* < 0.001	194	0.253/0.262 (CI 0.215 to 0.309) RR(exposed/unexposed) = 7.8 (CI 6.0 to 10.3), *P* < 0.001
No	10,877	154	0.014/0.014 (CI 0.009 to 0.019)	226	0.021/0.022 (CI 0.016 to 0.028)	345	0.032/0.033 (CI 0.025 to 0.042)

^a^GEE logistic regression model using Stata was fitted for GTI outcome with seasonality, triplet and homologous reported exposure (same or previous week) as covariates. The working correlation matrix was ‘independent’ and chosen by the quasi-likelihood model criterion (QIC).

^b^95% confidence interval.

^c^RR, risk ratio.

The relative risk of GTI associated with an exposure reported only-not-at-work during the same week was somewhat higher than that of an exposure reported ‘only at work’ (RR 1.5, CI 1.0 to 2.0) (Table 4). During the week following the reported exposure, the difference between the two categories of the exposure sites was not significant (Table 4). The risk associated with person weeks with an exposure experienced both at work and not-at-work was, unlike the situation with RTI, not higher than that associated with a reported exposure in either of the 2 category sites only, but between the 2 values, scoring 12.4 (CI 6.3 to 24.5) for the reported same week symptoms. For the following week symptoms the risk due to the reported double-site exposure, 4.6 (CI 1.9 to 11.0), appeared higher than that associated with a reported exposure at either site alone. However, the difference did not reach statistical significance (RR (WE/E) = 1.50 (CI 0.20 to 2.79).

**Table 4:** **Association of reported homologous exposure with reported gastrointestinal tract symptoms during the same or the following week including comparisons between sites of exposure**

**Reported exposure**		**Reported symptoms: Person-week in relation to exposure/Number and proportion of weeks with GTI symptoms/Statistical analysis**					
**Site**	**Number**	**Same week**		**Following week**		**Either week**	
		**Number**	**Proportion/predictive margin**	**Number**	**Proportion/predictive margin**	**Number**	**Proportion/predictive margin**
Only at work (W)	261	41	0.157/0.162 (CI^a^ 0.116 to 0.208) RR(W/0)^b^ = 11.4 (CI 7.5 to 17.5), *P* < 0.001	14	0.054/0.572 (CI 0.0290 to 0.0854) RR(W/0) = 2.6 (CI 1.6 to 4.4), *P* < 0.001	51	0.195/0.201 (CI 0.144 to 0.258) RR(W/0) = 6.0 (CI 4.2 to 8.6), *P* < 0.001
Only elsewhere (E)	437	109	0.249/0.246 (CI 0.193 to 0.298) RR(E/0) = 17.4 (CI 11.8 to 25.7), *P* < 0.001 RR(E/W) = 1.5 (CI 1.0 to 2.0)	48	0.110/0.0674 (CI 0.046 to 0.089) RR(E/0) = 3.1 (CI 2.2 to 4.3), *P* < 0.001 RR(E/W) = 1.18 (CI 0.52 to 1.84)	131	0.300/0.311 (CI 0.248 to 0.375) RR(E/0) = 9.0 (CI 6.8 to 11.9), *P* < 0.001 RR(E/W) = 1.5 (CI 1.0 to 2.0)
Both W and E	69	9	0.130/0.176 CI 0.053 to 0.299) RR(WE/0) = 12.4 (CI 6.3 to 24.5), *P* < 0.001	5	0.072/0.101 (CI 0.013 to 0.188) RR(WE/0) = 4.6 (CI 1.9 to 11.0), *P* < 0.001	12	0.174/0.186 (CI 0.049 to 0.322) RR(WE/0) = 5.5 (CI 1.5 to 9.7), *P* < 0.001

GEE logistic regression model using Stata was fitted for GTI outcome with seasonality, triplet and homologous reported exposure (same or previous week) as covariates. The working correlation matrix was ‘independent’ and chosen by the quasi-likelihood model criterion (QIC).

^a^95% confidence interval.

^b^RR, risk ratio, where 0 = No exposure from Table 3.

### Association of self-reported RTI and GTI episodes with self-recognized homologous exposure during the same or the preceding person week

We also studied the association of individual distinct RTI or GTI episodes with reported homologous exposure during the same or the preceding week. Unfortunately, we could not fit this ‘retrospective’ analysis in the statistical models available. Therefore, the following figures are only descriptive. About three quarters of the person weeks with a reported RTI episode (739/974) appeared to be associated with a reported exposure to RTI during the same or the preceding person-week. On the other side, about one quarter of the reported episodes (235/974) emerged without a recognized exposure during either week (Table 5). Again, the exposures outside the work appeared to be more frequent than those recorded ‘only at work’ (311 versus 179 for the same week). In this part describing the data broken down by the exposure site the either week aspect was omitted from the calculations because of mixtures: for example, exposure occurring ‘only at work’ during the same week and ‘only elsewhere’ during the preceding week would have complicated the analysis.

**Table 5:** **Self-reported distinct respiratory (RTI) and gastrointestinal tract infection episodes (GTI) broken down according to reported exposure during the same or the preceding week**

**Reported episode**		**Self-reported homologous exposure: site and timing**						
**Type**	**Total number**	**Site**	**Same week**		**Preceding week**		**Either/neither** ^**a**^ **week**	
			**Number**	**Proportion** ^**b**^	**Number**	**Proportion**	**Number**	**Proportion**
RTI	974	Anywhere	659	0.68	472	0.49	739	0.76
		None	315	0.32	502	0.52	235	0.24^a^
		Only at work (W)	179	0.18	137	0.14	NR	NR
		Only elsewhere (E)	311	0.32	224	0.23	NR	NR
		Both W and E	169	0.26	111	0.24	NR	NR
GTI	233	Anywhere	129	0.55	49	0.21	140	0.60
		None	104	0.45	184	0.79	93	0.40^a^
		Only at work (W)	37	0.16	12	0.05	NR	NR
		Only elsewhere (E)	89	0.38	33	0.14	NR	NR
		Both W and E	3	0.013	4	0.017	NR	NR

^a‘^Neither’ refers to the cells of the second and the seventh lines only and equals to no exposure recognized during either week.

^b^Proportion is calculated with reference to all person weeks with symptoms of RTI or GTI, respectively.

NR, not recorded.

A potential source of reported GTI infections appeared to be somewhat less obvious with about 60% of self-reported GTI episodes (140/233) being associated with a reported homologous exposure during the same or the preceding person week (Table 5). As in the case of RTI episodes, more GTI-associated homologous exposures had been reported outside the work than ‘only at work’ during both weeks (89 versus 37 and 33 versus 12, respectively).

## Discussion

This study assessed the temporal association of self-reported exposures to persons with symptoms of RTI or GTI with self-reported occurrence of homologous symptoms in the respondent during the same or the following week. An exposure to RTI was reported during more than a quarter of all follow-up weeks while that to GTI was noted in less than one tenth of the weekly reports. Reported exposure to either type of infection clearly increased the relative risk for homologous symptoms in the respondents during both the same week and the following week. The relative risk for homologous disease was higher in persons exposed to GTI than to RTI.

General population-based studies trying to figure out a quantitative relationship between a homologous recognized exposure and subsequent onset of an acute RTI or GTI are difficult to find in the literature. Our current attempt to investigate the matter by the implemented weekly self-reporting principle can be envisaged to have some obvious weaknesses. Firstly, the data collection was by self-reporting of RTI and GTI symptoms, as well as of the exposures, by lay persons rather than through objective assessment of symptoms and signs by health care professionals. In theory, this might decrease the accuracy and consistency of the collected data although the relevant definitions were repeated weekly in the personal Emails requesting the report. Seasonal variation of the monthly rate of the reported exposures was similar to that of the reported onsets of infection episodes in the study population, which was previously found to well reflect the seasonal variation of the epidemic activity of common viral infections in the source population [12]. This suggests that most exposure reports could be relevant but with the reservations mentioned below.

A second problem is the fact that while the occurrence of RTI and GTI symptoms was reported on a daily basis, the recognized exposures were scored only on a weekly basis [11]. As a large proportion of the infections were reported to start on Monday or Tuesday [15] an exposure reported for the same week may have occurred either before or after the onset of the reported infection and, therefore, we cannot use the observed exposure-infection associations for considering potential causative relationship between the two events. Rather, exposures reported for the same week as the onset of infection reflect the overall activity of infection transmission around the reporting participant. On the other hand, because the incubation period of many viral RTIs and GTIs may be as short as 1 to 2 days [16], and because the duration of a large part of both type of infections in this study was less than 4 days [15], a putative exposure on Monday may have resulted in a short-duration disease not continuing over the following weekend, and thus might no longer be reported in the following week’s report. Hence, the same week’s associations are likely to give somewhat falsely high figures while the successive week’s associations may result in falsely low figures. Therefore, the described associations of reported exposures with RTI or GTI symptoms reported for either of the 2 weeks must be interpreted with caution as regards the exact numbers.

Although a weekly electronic reporting principle implemented in this study has been considered feasible for epidemiological studies on common infectious diseases [17], it is clear that daily diaries of both symptoms and recognized exposures would have minimized the recall bias [17]. We did not use daily symptom diaries because, when designing the data collection system for the STOPFLU trial, we wanted to create an electronic weekly reporting system that was so simple and easy to use, that the trouble of filling it would not be a reason for dropout and not to continue reporting for the desired more than 1-year duration of the trial [11]. This goal was reached, but as a compromise, we had to limit the details of the data to be collected. An additional reason for choosing this retrospective only-weekly reporting of exposures was our consideration that while it is likely that a person will remember the day of a personal disease onset for 7 days, it is much less probable that he or she can reliably list exposures on a daily basis in the weekly report.

In both types of infections, most of the reported homologous exposures were recorded for the same week as the self-reported symptoms. We cannot exclude the possibility that part of this difference is due to recall bias, as it is a natural behavior for humans to try to remember a potential source for his own infection, whereas it would be easier to forget exposures concerning weeks without symptoms. As for the generalizability of our results, a further note of caution may be necessary. In addition to the inherent differences between a study set-up and normal life, and on top of the above reservations, one has to remember a potential selection bias in our study. Only a proportion of the staff in the study clusters participated in this study, and we have no way to assess how well they represented the entire staff.

A temporal association between an exposure to RTI or GTI and homologous infection cannot be considered big news, especially with the above reservations, but some figures in our results are worth commenting on. The duration of the trial was 16 months, which means that both reported exposures and infections covered a wide range of different causative agents, as we believe, mostly viruses showing different types of seasonal variation of transmission. Seasonality, randomization triplet, cluster effects, and longitudinal associations among observations of each subject were taken into account in our statistical analysis of the exposure-infection relationships. The overall occurrence of reported exposures to RTI was 4 to 5 times higher than that to GTI, a similar ratio as in the reported infection episodes. While a recognized exposure to RTI was associated with reported homologous symptoms in the respondent during the same or the following week more frequently than in the case of GTI, a calculated relative risk of disease associated with an observed exposure was higher for GTI than for RTI. This was based on the much lower rate of GTI infections, as compared to RTI, occurring in association with person weeks without a reported exposure. A possible explanation for the greater relative risk of a homologous disease associated with a reported GTI exposure, as compared to that with an RTI exposure, may be that adults recognized by an outsider to have symptoms of GTI are relatively more infectious than those with an obvious RTI. On the other hand, based on the descriptive assessment of the data the other way around, almost a quarter of the reported designated RTI episodes and as many as about 40% of the GTI episodes occurred without a personal recognition of a homologous exposure during the same or the preceding week. Many viruses are known to be shed by infected persons without clear concomitant symptoms of disease. The difference between RTI and GTI is in line with the fact that RTI symptoms in adults are usually more apparent to outsiders than those of GTI.

Significantly more exposures to both diseases were reported having occurred only-not-at-work than ‘only at work’ and the relative risks of disease associated with the outside work exposures were greater than those occurring ‘only at work’. A possible explanation is that contacts with sick family members are likely to be more intimate, long-lasting and unavoidable than those with symptomatic colleagues at work. Person weeks with recognized RTI exposure ‘both at work and elsewhere’ showed significantly higher relative risk for infection than those with exposures only at one site category. A straightforward explanation would be an additive probability of transmission due to a likely greater number of exposure events. For GTI a similar comparison did not reveal a statistically significant difference, but this may be because of the small number of recorded events.

## Conclusions

Self-reported exposure to RTI and, especially, that to GTI remarkably increased the relative risk of reported homologous disease symptoms in the respondents. On the other hand, both types of infection episodes also frequently occurred without an association to a reported exposure. Hence, social distancing as a means of control of both RTI and GTI epidemics may be justified, and should then include both avoiding contacts with persons with RTI or GTI symptoms and people gatherings in general. Likewise, effective hand hygiene can be recommended in support of social distancing through epidemic seasons even in the absence of self-recognized exposures. Reported exposures outside the work appeared to significantly contribute to RTI or GTI in office workers and hence, any intervention aiming at reducing occupational infections should be implemented with the 24 hours a day 7 days a week principle and also cover the free time in order to be effective.

## Abbreviations

CI: confidence interval
GEE: generalized estimating equations
GTI: gastrointestinal tract infection
KTL: National Public Health Institute, Finland (predecessor of THL)
MCAR: missing completely at random
QIC: quasi-likelihood model criterion
QICC: corrected quasi-likelihood under independence model criterion
RR: risk ratio
RTI: respiratory tract infection
SD: standard deviation
THL: National Institute for Health and Welfare, Finland
